# Partial Enteral Nutrition Preserves Elements of Gut Barrier Function, Including Innate Immunity, Intestinal Alkaline Phosphatase (IAP) Level, and Intestinal Microbiota in Mice

**DOI:** 10.3390/nu7085288

**Published:** 2015-08-03

**Authors:** Xiao Wan, Jingcheng Bi, Xuejin Gao, Feng Tian, Xinying Wang, Ning Li, Jieshou Li

**Affiliations:** 1 Department of General Surgery, Jinling Hospital, School of Medicine, Nanjing University, Nanjing 210002, China; E-Mails: van395412495@sina.cn (X.W.); ahbijingcheng@163.com (J.B.); xuejingao870214@163.com (X.G.); easyhard666@163.com (F.T.); ninglijinling@163.com (N.L.); lijieshouarmy@yeah.net (J.L.); 2 Department of General Surgery, South Medical University, Guangzhou 510515, China

**Keywords:** partial enteral nutrition, gut barrier, intestinal alkaline phosphatase, innate immunity, intestinal microbiota

## Abstract

Lack of enteral nutrition (EN) during parenteral nutrition (PN) leads to higher incidence of infection because of gut barrier dysfunction. However, the effects of partial EN on intestina linnate immunity, intestinal alkaline phosphatase (IAP) and microbiota remain unclear. The mice were randomized into six groups to receive either standard chow or isocaloric and isonitrogenous nutritional support with variable partial EN to PN ratios. Five days later, the mice were sacrificed and tissue samples were collected. Bacterial translocation, the levels of lysozyme, mucin 2 (MUC2), and IAP were analyzed. The composition of intestinal microbiota was analyzed by 16S rRNA pyrosequencing. Compared with chow, total parenteral nutrition (TPN) resulted in a dysfunctional mucosal barrier, as evidenced by increased bacterial translocation (*p* < 0.05), loss of lysozyme, MUC2, and IAP, and changes in the gut microbiota (*p* < 0.001). Administration of 20% EN supplemented with PN significantly increased the concentrations of lysozyme, MUC2, IAP, and the mRNA levels of lysozyme and MUC2 (*p* < 0.001). The percentages of Bacteroidetes and Tenericutes were significantly lower in the 20% EN group than in the TPN group (*p* < 0.001). These changes were accompanied by maintained barrier function in bacterial culture (*p* < 0.05). Supplementation of PN with 20% EN preserves gut barrier function, by way of maintaining innate immunity, IAP and intestinal microbiota.

## 1. Introduction

Parenteral nutrition (PN) prevents progressive malnutrition and provides lifesaving therapy for many patients with gastrointestinal disorders. However, PN is associated with an increased incidence of infection in critically ill patients [1,2,3]. For the past 20 years, many investigators have focused on this serious issue, and several studies have suggested that impairment of intestinal barrier function might be at least partially responsible [4,5].

The intestinal barrier is composed of several components. Innate immunity, comprising a series of nonspecific antibacterial components, plays an important role in this system. The goblet cells release a variety of glycoproteins, such as mucin 2 (MUC2), to form a mucin layer that covers the inner surface of the intestine. This protein layer functions as a natural protective barrier against harmful antigens and pathogens. Paneth cells are located in the crypt and function primarily in the secretion of several kinds of antimicrobial peptides, including lysozyme [6]. Previous studies have shown that PN without intestinal stimulation significantly impairs innate mucosal immune function by reducing the quantity of goblet cells and concentration of lysozyme and MUC2 in the intestine [7].

Intestinal alkaline phosphatase (IAP) is another important component of the intestinal barrier. IAP, a glycoprotein anchored in the apical membrane by a glycosyl-phosphatidyl-inositol linkage, plays multiple roles in the maintenance of the intestinal barrier, including regulation of pH at the duodenal surface, detoxification of bacterial lipopolysaccharides and free nucleotides, reduction of intestinal inflammation, and modulation of gut microbiota [8,9,10]. It also can inhibit the expression of vascular endothelial growth factor (VEGF), which may increase intestinal permeability in order to reduce bacterial translocation [11]. In addition, gastrointestinal administration of exogenous IAP ameliorates gut inflammation, favors gut tissue regeneration, and reverses the gut barrier dysfunction and tight junction protein losses due to a lack of enteral feeding [10,12]. In addition, intense research aimed at discovering effective therapeutic strategies to reverse gut barrier dysfunction is currently ongoing.

The intestinal microbiota, which also refers to the biological barrier, also plays an indispensable role in maintaining intestinal barrier function. It collectively consists of 10^14^ colony-forming units of bacteria, *i.e.*, ten-fold the number of cells in a human. Gut microbes play an important role in host immune stimulation and maintenance, digestion, and production of short-chain fatty acids and vitamins [13,14]. In a previous study, when compared with chow-fed mice, the microbiome of PN-fed mice was shown to contain reduced levels of bacteria from the phylum Firmicutes and increased levels of bacteria from the phyla Bacteroidetes and Proteobacteria [5].

Because PN is associated with intestinal barrier damage leading to poor clinical outcomes, research is currently focused on mechanisms for effective protection of the intestinal barrier [15,16]. The use of enteral nutrition (EN), also known as trophic feeding, can protect the intestinal barrier; however, because of gastrointestinal intolerance, approximately one-third of patients cannot tolerate the full dose of EN in the initial phase [17,18]. Moreover, whether the intestinal barrier can be sufficiently protected by the administration of EN is not currently clear. Clinical studies have confirmed that a small amount of EN result in similar clinical outcomes as full-energy EN but with fewer episodes of gastrointestinal intolerance [19,20]. A retrospective analysis also revealed that patients who obtained more than 10% of their total calories via enteral feeding had better clinical outcomes than those receiving total parenteral nutrition (TPN) [21]. However, whether these improved clinical outcomes are associated with maintenance of intestinal barrier function is unclear. Animal experiments have demonstrated that when EN accounts for 20%–30% of the total calorie intake, levels of intestinal secretory immunoglobulin A (a component of acquired immunity) are maintained, the morphology of intestinal villi and crypts are protected, and bacterial translocation decreases [22,23,24]. These findings suggest that the function of the damaged intestinal barrier could be partially protected upon administration of EN; however, the lowest dose required to protect the gut barrier has yet to be established. In addition, the effects of partial EN on innate immunity, IAP levels, and intestinal microbiota are currently unknown. Therefore, in this study, we investigated the effect of partial EN on elements of intestinal barrier function, including innate immunity, IAP levels, and intestinal microbiota. Our results provide insights into the required threshold dose for EN, as well as the mechanisms underlying the protective effects of partial EN on the intestinal barrier in a mouse model.

## 2. Materials and Methods

### 2.1. Animals

The study was approved by the Animal Care and Use Committee of Nanjing University and Jinling Hospital (MARC20130712001, 12 July 2013), Nanjing. Male, outbred Institute of Cancer Research mice were purchased from the Laboratory Animal Research Center of Jiangsu University (Zhenjiang, China). They were fed standard mouse chow (Rodent Diet 5001, LabDiet; PMI Nutrition International, St. Louis, MO, USA) and water, and maintained in conditions with controlled temperature, humidity, and light cycle (12/12-h light/dark cycle) for 1 week (acclimatization) prior to initiation of the study protocol.

### 2.2. Study Design

Sixty male mice (6–8-weeks-old) weighing 25–30 g were randomized to receive either standard chow (*n* = 10), TPN (*n* = 10), or 10%, 20%, 40%, or 60% partial EN supplemented with PN (*i.e.*, 10% EN + 90% PN, 20% EN + 80% PN, 40% EN + 60% PN, 60% EN + 40% PN; *n* = 10 per group). The animals were anesthetized by intraperitoneal administration of ketamine (100 mg/kg body weight) [5]. Their neck and mid-scapular regions were shaved and prepared with povidone iodine. Afterwards, the external jugular vein was isolated, and a silicone rubber catheter (0.305 mm inner diameter, 0.635 mm outer diameter; Helix Medical Inc., Carpentaria, CA, USA) was placed into the vein for intravenous infusion. The distal end of the catheter was tunneled subcutaneously over the back to pierce the midpoint of the tail. The mice were partially restrained by the tail; this method of restraint does not induce significant stress [5,7,25].

After catheter placement, 0.9% saline was infused into each mouse at 4 mL/day for 2 days after surgery, and *ad libitum* water and chow were provided. Subsequently, the mice in the TPN and partial EN + supplemental PN (EN + PN) groups received the appropriate solution at 4.4 mL/day (day 1), 7.7 mL/day (day 2), and 11 mL/day (days 3–5) along with *ad libitum* water throughout the study. The mice in the chow group received 4 mL/day intravenous 0.9% saline along with free access to chow and water. The formulation of the TPN solution has been described previously [5,7]. Briefly, it contained 5.3% amino acids, 32% dextrose, electrolytes, and multivitamins at 1280 kcal/L, and a non-protein calories/nitrogen ratio of 149:1 [5,7]. The 10%, 20%, 40%, and 60% EN solutions were formulated with 0.31 g, 0.62 g, 1.24 g, and 1.86 g, Nutren^®^ powder, respectively; Nutren^®^ powder contains 15.9% amino acids, 57.4% carbohydrates, 14.0% lipids, electrolytes, and multivitamins, with a non-protein calorie/nitrogen ratio of 130.4:1 (545.1 kJ/g nitrogen). The formulations were calculated according to the percentage of calories they contained. The entire dose of powder was administered every day during the experiment. The TPN and EN + PN formulations were almost isocaloric and isonitrogenous, and they met the calculated nutrient requirements of mice weighing between 25–30 g [5,7]. After 5 days of feeding (*i.e.*, 7 days after catheterization), the mice were anesthetized as described above and sacrificed. The small intestine was removed and flushed with 20 mL of buffer (Hank’s Balanced Salt Solution; Bio Whittaker, Walkersville, MD, USA). One microliter of wash fluid was stored for investigation of intestinal microbiota. The mesenteric lymph nodes, liver, and portal venous blood were collected aseptically for bacterial culture from the mice. Three-centimeter segments of ileal tissue samples, including the Peyer’s patches, were resected and stored at −80 °C for subsequent analysis. For immunohistochemistry (IHC) analysis, the tissue samples were treated with 4% paraformaldehyde overnight, and then transferred to 70% ethanol and stored at 4 °C until subsequent analysis.

### 2.3. Bacterial Culture

Tissue samples (0.3 g) were obtained aseptically, weighed, and then homogenized in nine-times their weight of phosphate-buffered saline (PBS). Each dilution (0.2 mL) was plated on blood agar (Difco, BD, Franklin Lakes, NJ, USA) and incubated at 37 °C for 48 h. Colony forming units were examined to determine whether bacterial translocation had occurred in the animals.

### 2.4. Periodic Acid–Schiff Staining

Ileal tissue sections were processed (Tissue-Tek^®^ VIP^®^; Sakura Finetek, Japan) and fixed in paraffin wax. The tissue sections were cut into 5-µm-thick slices, deparaffinized, rehydrated through graded ethanol washes (2 min each of 100% ethanol, twice, 95% ethanol, twice, 70% ethanol, once), and placed into distilled H_2_O. The samples were then stained with periodic acid-Schiff stain (ab-150680; Abcam, Cambridge, UK).The number of goblet cells was determined by counting the average number of goblet cells per field of vision in representative active microscopic fields (original magnification = ×40) per mouse. The histomorphometric measurements were conducted by two independent, blinded researchers (Xiao Wan and Xuejin Gao).

### 2.5. IHC for Lysozyme and IAP in Ileal Tissue Samples

The ileal tissue sections were processed (Tissue-Tek^®^ VIP^®^; Sakura Finetek) and fixed in paraffin wax. The wax blocks were cut into 5-µm-thick slices and deparaffinized. Antigen retrieval was performed in sodium citrate buffer. The sections were blocked in 5% bovine serum albumin (BSA) in PBS for 1 h, and then incubated with the primary antibody overnight at 4 °C. Subsequently, the sections were processed using the DAB Detection Kit (SP-9000-D; ZSGB-Bio, Beijing, China) according to the manufacturer’s protocol. Hematoxylin staining was used to counterstain nuclei. The IHC features shown in the figures are representative of all tissue samples studied. The primary antibody are as following: lysozyme (ab-108508; Abcam; 1:1000 dilution); IAP (ab-108337; Abcam; 1:250 dilution).

### 2.6. Western Blot Analysis for Lysozyme, MUC2 and IAP in Ileal Tissue Samples

Total tissue protein (80 µg) extracted from the ileal tissue samples was used to quantify. The protein sample was heated at 95 °C for 5 min, and then separated on a polyacrylamide gel by electrophoresis at 20 mA until the bands were separated, before being transferred to a polyvinylidene fluoride (PVDF) membrane (pore size: 0.45 µm) using the standard transfer buffer. After washing for 5 min in Tris-buffered saline with 1% Tween^®^-20 (TBS-Tween), the membranes were blocked in BSA for 1 h and then incubated with the primary antibody overnight at 4 °C. Following incubation, the membranes were washed and further incubated with the secondary antibody for 1 h at room temperature with constant agitation. After washing, the membranes were incubated with electrochemiluminescence solution for 5 min, and the bands were detected using photographic film. Bands corresponding to a predicted molecular weight were used to verify targeted protein. Glyceraldehyde-3-phosphate dehydrogenase (GAPDH) was used as an internal control to normalize the density among multiple membranes. The key points of WB for each protein are shown in Table 1.

**Table 1:** Key points of Western blot analysis for lysozyme, MUC2 and IAP in ileal tissue samples.

Protein	Lysozyme	MUC2	IAP
electrophoresis	20 mA; 120 min	20 mA; 135 min	20 mA; 120 min
% of SDS	15%	5%	10%
transferring	100 V; 60 min	100 V; 180 min	100 V; 80 min
primary antibody	ab-108508; Abcam; 1:5000 dilution in BSA	ab-11197; Abcam; 1:1250 dilution in BSA	ab-108337; Abcam; 1:10,000 dilution in BSA
Secondary antibody	A9169; 1:10,000 dilution in BSA	31439; 1:10,000 dilution in BSA	A9169; 1:10,000 dilution in BSA

IAP: intestinal alkaline phosphatase; MUC2: mucin 2; BSA: bovine serum albumin; SDS: sodium dodecyl sulfate.

### 2.7. Quantitative Polymerase Chain Reaction (qPCR) Analysis for Lysozyme and MUC2

The frozen distal ileal tissue samples were ground in RNAiso Plus (#9108; Takara Bio, Dalian, China). Samples were processed according to the manufacturer’s protocol, and RNA was extracted. The purity and concentration of RNA was determined by measuring the optical density at 260 and 280 nm. One microgram of RNA was used as the template for reverse transcription, with random primers and reverse transcriptase used in a cDNA synthesis reaction according to the manufacturer’s instructions (RR047A; Takara Bio). SYBR^®^ Green Select Master Mix was used to perform qPCR. Primers were designed and synthesized by Invitrogen (Carlsbad, CA, USA). The mRNA expression level of lysozyme was determined by using the software provided with the 7500 Fast Real-Time PCR system (Applied Biosystems, Waltham, MA, USA) and the Δ*C*_t_ relative quantification model. Relative expression was calculated using the 2^−ΔΔ*Ct*^ method after values were normalized against those of GAPDH. The geometric mean of the GAPDH expression level was used as the normalization factor. The sequences of the primers were as follows: lysozyme, 5′-ATGGCGAACACAATGTCAAA-3′ and 5′-GCGAGGAAGTGTGACCTCTC-3′; MUC2, 5′-ACAAAAACCCCAGCAACAAG-3′ and 5′-GAGCAAGGGACTCTGGTCTG-3′. IAP, 5′-CTCATCTCCAACATGGAC-3′ and 5′-TGCTTAGCACTTTCACGG-3′.

### 2.8. Sample Collection and DNA Isolation

A distal ileal tissue sample (1-cm in length) was dissected from each mouse. The ileal tube was flushed with 1 mL of Hank’s Balanced Salt Solution and the fluid was collected. Bacterial DNA was isolated from 0.2 mL of each sample using the PureLink™ Genomic DNA Mini Kit (K1820-00; Invitrogen).

### 2.9. 16S rRNA Pyrosequencing

The partial 16S rRNA sequence was obtained using the Ion16S™ Metagenomics Kit (A26216; Thermo Fisher Scientific, Waltham, MA, USA). The 5′-ends of the forward primers were fused with the A-Adaptor plus key sequence, whereas the reverse primers were fused with the truncated Pi-adapter sequence (trP1). The V3–V5 region, comprising approximately 400 base pairs (bp) of the 16S rRNA gene, was selected to construct a community library by tag-encoded pyrosequencing. The broadly conserved primers 517F (5′-GCCAGCAGCCGCGGTAA-3′) and 926R (5′-CCGTCAATTYYTTTRAGTTT-3′) were used to amplify this region. The resulting products were quantified using a NanoDrop™ systemand a Qubit^®^ fluorometer (both Invitrogen) prior to sequencing. Sequencing of the amplicon libraries was conducted using the Ion Torrent Personal Genome Machine (PGM) system with the Ion PGM™ Sequencing 400 Kit (4482002; Thermo Fisher Scientific, Waltham, USA) following the corresponding protocol (MAN0007242 Rev 1, 3 March 2013). The data-processing pipeline removed low-quality reads according to the following criteria: (1) reads that did not completely match the PCR primer and barcode; (2) reads that were shorter than 200 bp or longer than 500 bp; (3) reads that contained >3 undetermined nucleotides; and (4) reads with an average quality score < 20. The taxonomic status (phylum and genus) was assigned to each read using a parallelized version of the Greengenes database [5,26]. Operational taxonomic unit (OTU) analysis was conducted on a clustering basis for each sample. Readswere clustered using the complete linkage-clustering algorithm implemented with a 97% identity threshold [27]. Rarefaction curves were calculated by using RarefactWin software [28]. The species richness estimator Chao1, and the Shannon and Simpson diversity indices, were calculated using Quantitative Insights Into Microbial Ecology (QIIME) [29].

### 2.10. Statistical Analysis

All data are reported as means ± standard error of the mean (SEM). All statistical analyseswere conducted using SPSS 20.0 (IBM, New York, NY, USA), and statistical significance (accepted at *p* < 0.05) was determined using a fixed-effects analysis of variance model with the Fisher protected least significant difference *post hoc* test.

## 3. Results

### 3.1. Bacterial Culture

As shown in Table 2, 80% of the mesenteric lymph nodes from mice administered TPN tested positive for bacteria compared with 10% (1/10) in the chow group (*p* < 0.05). The positive rate of bacterial culture was significantly lower in mice administered with over 20% EN than those in the TPN group. Only occasional bacterial translocation was observed in the liver or portal blood, and no significant differences were achieved.

**Table 2:** Incidence of bacterial translocation in the mice, as estimated by tissue bacterial culture.

Incidence of Bacterial Translocation	Chow	10% EN	20% EN	40% EN	60% EN	TPN
Mesenteric lymph node	10% (1/10)	60% (6/10) ^a^	30% (3/10) ^b^	0 (0/10) ^b^	10% (1/10) ^b^	80% (8/10) ^a^
Liver	0 (0/10)	10% (1/10)	0( 0/10)	0 (0/10)	0 (0/10)	10% (1/10)
Portal venous blood	0 (0/10)	10% (1/10)	10% (1/10)	0 (0/10)	0 (0/10)	10% (1/10)

Data were presented as positive rate (number of positive bacterial culture/total number of bacterial culture).^a^ *p* < 0.05 *vs.* chow; ^b^ *p* < 0.05 *vs.* total parenteral nutrition (TPN), as calculated by chi-square analysis; PN, parenteral nutrition; EN, enteral nutrition.

### 3.2. Quantity of Goblet Cells

**Figure 1:**
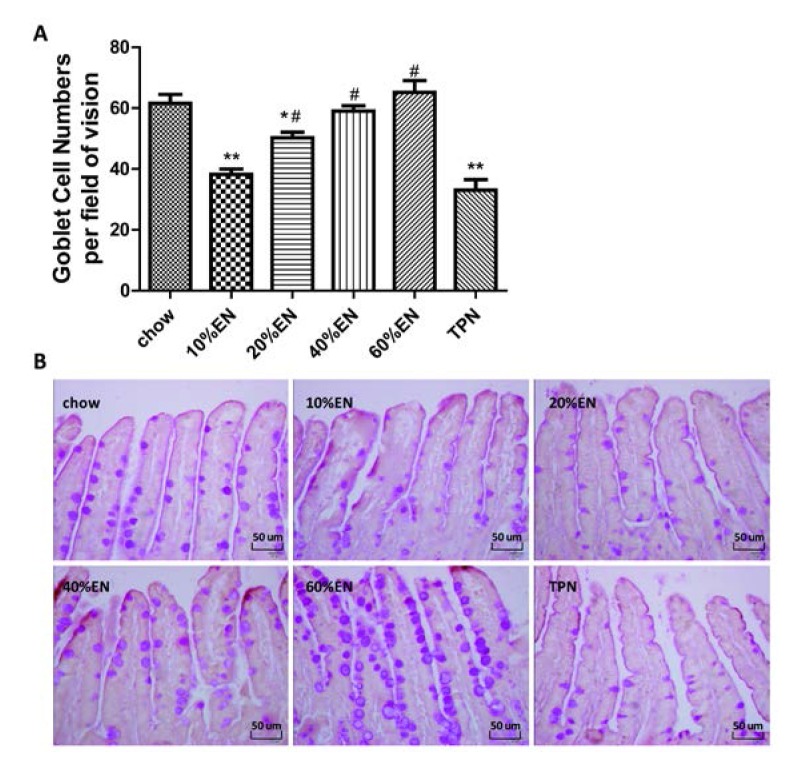
Morphological changes in the ileal tissue in each treatment group. (**A**) Numbers of goblet cells per field of vision; Data are presented as the mean ± standard error of the mean (SEM). ** *p* < 0.001 *vs.* chow. * *p* < 0.05 *vs.* chow. ^#^ *p* < 0.001 *vs.* TPN; (**B**) Representative images of periodic acid-Schiff (PAS) base-stained paraffin sections in each group. PAS-stained goblet cells are visible in the epithelial layer (original magnification: ×40). EN: enteral nutrition; TPN: total parenteral nutrition.

The number of goblet cells per field of vision was significantly lower in the TPN group compared with the chow group (*p* < 0.001). Supplementation with EN at a dose of 20% resulted in a greater number of goblet cells compared with TPN treatment; however, this value remained lower than the quantity of goblet cells observed in mice from the chow group (*p* < 0.05). Administration of both 40% and 60% EN led to complete recovery of goblet cell number to a chow level. The average number of cells per group is shown in Figure 1 and Table S1.

### 3.3. Lysozyme Levels in the Ileal Tissue Samples

**Figure 2:**
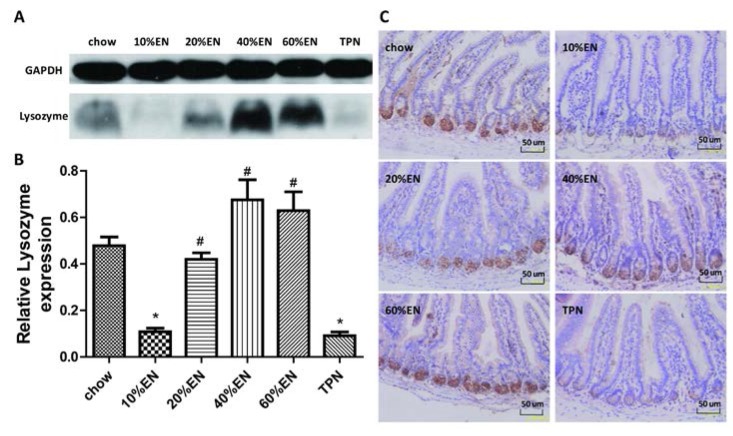
Lysozyme levels in the ileal tissue. (**A**) Lysozyme bands, as detected by Western blot analysis; (**B**) Lysozyme levels in the ileal tissue. Data are presented as the mean ± standard error of the mean (SEM). * *p* < 0.001 *vs.* chow. ^#^ *p* < 0.001 *vs.* TPN; (**C**) Immunohistochemical analysis of lysozyme in each group. Original magnification: ×40. EN: enteral nutrition; TPN: total parenteral nutrition; GAPDH: Glyceraldehyde-3-phosphate dehydrogenase.

Western blot analysis revealed that lysozyme levels in the ileal tissues of mice from the TPN group were significantly lower than those in the chow group (*p* < 0.05). The level of tissue lysozyme in the 10% EN group was similar to that in the TPN group; however, the tissue level of lysozyme was four-fold greater in the 20% EN group than in the TPN group (*p* = 0.01), with a value that was similar to that in the chow group. Lysozyme levels observed in the 40% EN and 60% EN groups were also similar to those in the chow group, but significantly higher than levels in the TPN group (Figure 2A,B and Table S2). The results of IHC analysis for lysozyme were consistent with those of Western blot analysis (Figure 2C).

### 3.4. IAP Levels in the Ileal Tissue Samples

**Figure 3:**
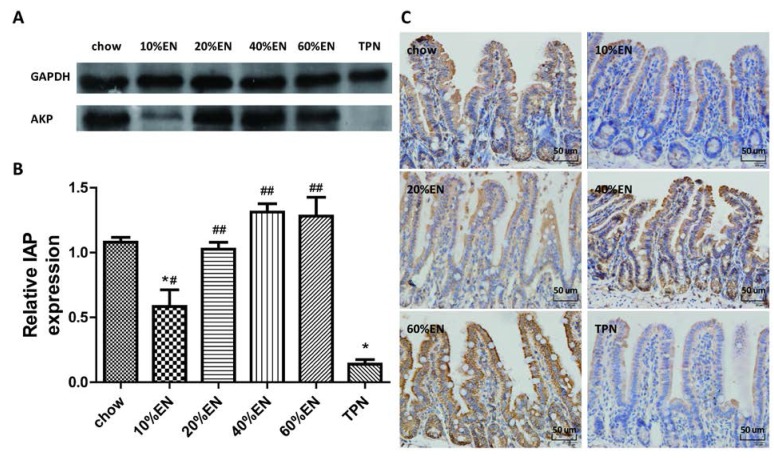
Intestinal alkaline phosphatase (IAP) levels in the ileal tissue. (**A**) IAP protein bands, as detected by Western blot analysis; (**B**) IAP levels in the ileal tissue. Data are presented as the mean ± standard error of the mean (SEM). * *p* < 0.001 *vs.* chow. ^#^ *p* < 0.05 *vs.* TPN. ^##^ *p* < 0.001 *vs.* TPN; (**C**) Immunohistochemical analysis of IAP in each group. Original magnification: ×40. EN: enteral nutrition; TPN: total parenteral nutrition; GAPDH: Glyceraldehyde-3-phosphate dehydrogenase.

A significantly lower levelof IAP was observed in the TPN group compared with the chow group (*p* < 0.001). Compared to TPN treatment, the addition of 10% EN enhanced the tissue level of IAP (*p* = 0.001), but this level remained lower than that observed in the chow group (*p* = 0.002). IAP levels in the 20% EN, 40% EN, and 60% EN groups were comparable to the levels in the chow group (*p* > 0.05). IHC analysis for IAP in the ileum revealed that PN reduced the expression of IAP in comparison to a chow diet. Furthermore, the overall expression level of IAP increased after 5 days administration of 20% EN (Figure 3 and Table S2).

### 3.5. MUC2 Levels in the Ileal Tissue Samples

**Figure 4:**
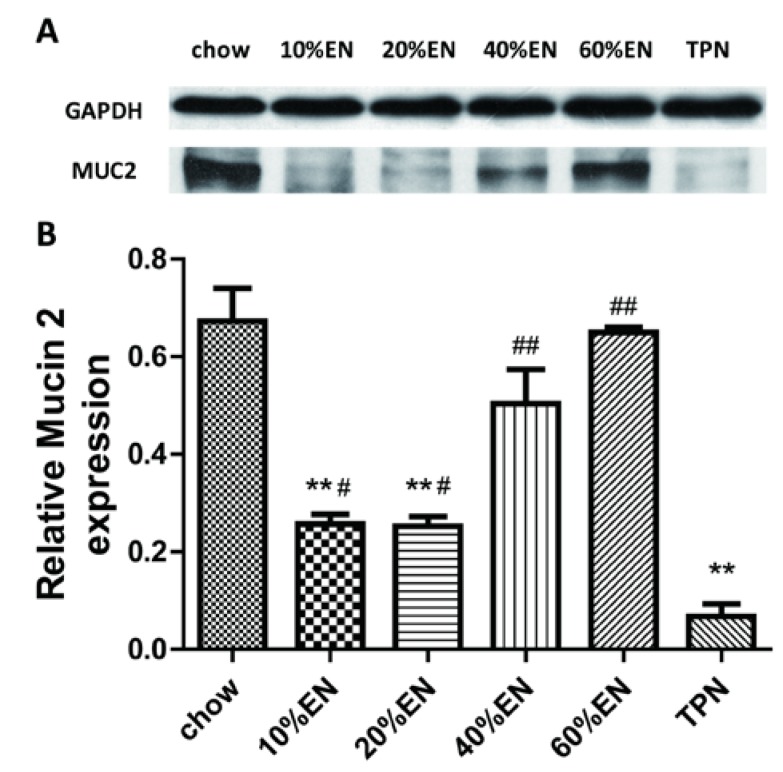
Relative density of mucin 2 (MUC2) in the ileal tissue. (**A**) Representative bands of MUC2, as detected by Western blotting; (**B**) MUC2 levels in the ileal tissue. Data are presented as the mean ± standard error of the mean (SEM). ** *p* < 0.001 *vs.* chow. ^#^ *p* < 0.05 *vs.* TPN. ^##^ *p* < 0.001 *vs.* TPN. EN: enteral nutrition; TPN: total parenteral nutrition; GAPDH: Glyceraldehyde-3-phosphate dehydrogenase.

The relative density of MUC2 in the ileal tissue was significantly lower in the TPN group compared with the chow group (*p* < 0.001). Consistent with changes in the quantity of goblet cells, MUC2 levels were significantly higher in the EN groups than in the TPN group (10% EN *vs.* TPN, *p* < 0.05; 20% EN *vs.* TPN, *p* < 0.05; 40% EN *vs.* TPN, *p* < 0.001; 60% EN *vs.* TPN, *p* < 0.001). However, with the exception of 60% EN, MUC2 levels in all EN groups remained lower than the level observed in the chow group (Figure 4 and Table S2).

### 3.6. Lysozyme mRNA Levels in the Ileum

**Figure 5:**
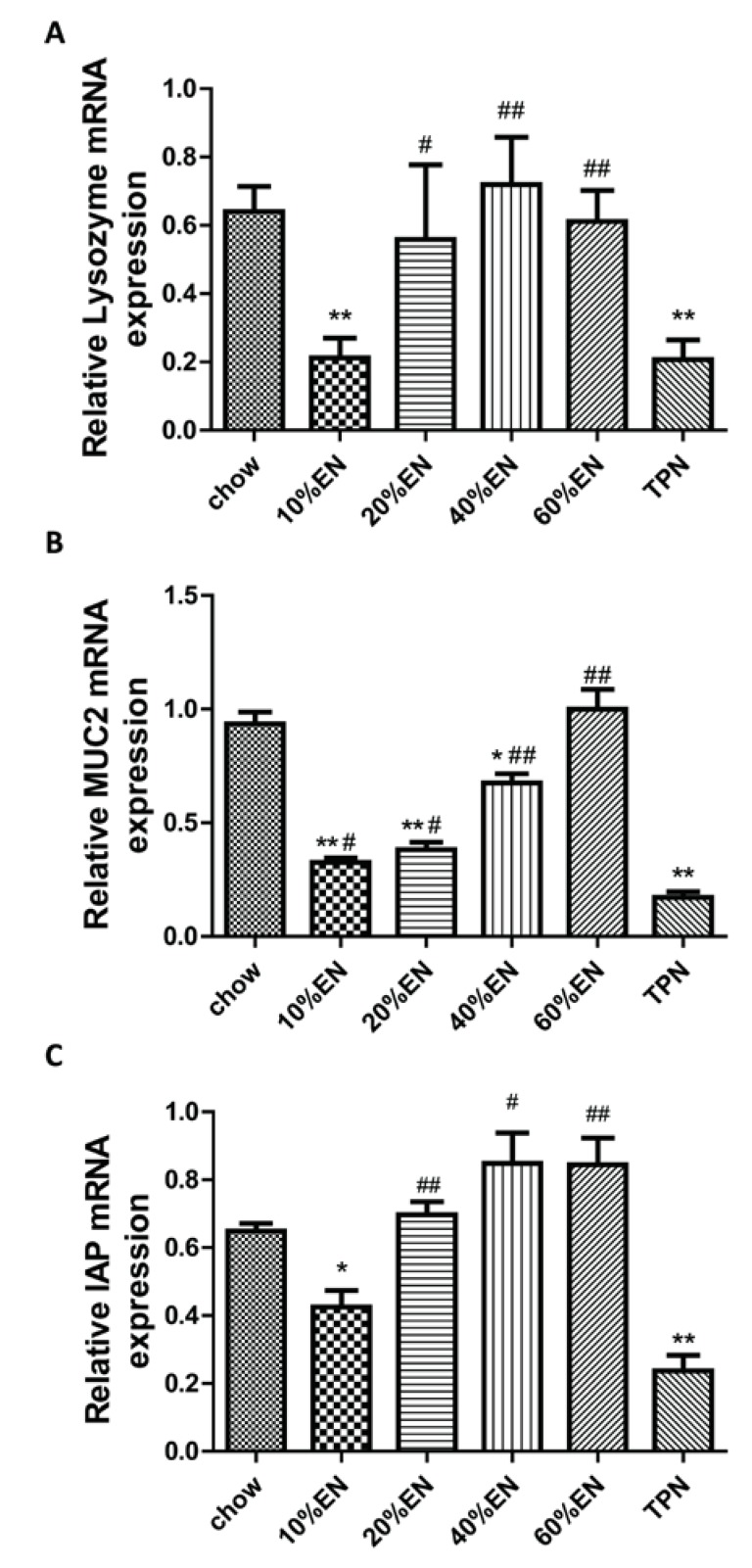
Reverse transcription quantitative polymerase chain reaction analysis of the expression levels of innate immunity products in the ileal tissue. (**A**) Lysozyme; (**B**) mucin 2 (MUC2) and (**C**) intestinal alkaline phosphatase (IAP). Data are represented as the mean ± standard error of the mean (SEM). * *p* < 0.05 *vs.* chow. ** *p* < 0.001 *vs.* chow. ^#^ *p* < 0.05 *vs.* TPN. ^##^ *p* < 0.001 *vs.* TPN. EN: enteral nutrition; TPN: total parenteral nutrition.

Consistent with the results of the protein-content analysis, qPCR analysis of lysozyme revealed thatrelative expression of lysozymewas significantly lower in the TPN groupcompared with the chow group (*p* = 0.013). In the 10% EN group, expression of lysozyme mRNA was lower than in the chow group (*p* < 0.05); however, the levels of lysozyme mRNA expression observed in the 20% EN, 40% EN and 60% EN groups were similar to that in chow groups (Figure 5A and Table S3).

### 3.7. MUC2 mRNA Levels in the Ileum

As shown in Figure 5B, the results of the qPCR analysis for MUC2 were consistent with the levels of MUC2 protein observed in the tissuesamples. MUC2 mRNA levels were significantly lower in the TPN group when compared withthe chow group (*p* < 0.001).Although the administration of partial EN ameliorated these effects to some extent, only 60% EN entirely maintained the levels of MUC2 mRNA to the extent observed in the chow group.

### 3.8. IAP mRNA Levels in the Ileum

Consistent with the results of the protein-content analysis, qPCR analysis of IAP revealed that relative expression of IAP mRNA was significantly lower in the TPN group compared with the chow group (*p* < 0.001). After the administration of 10% EN, the level of IAP mRNA was still lower than that in the chow group (*p* < 0.05). However, no significant difference of IAP mRNA expression could be observed in mice, which acquired more than 20% EN when they were compared with the chow group (Figure 5C).

### 3.9. 16S rRNA Pyrosequencing of Mouse Ileum Digesta

**Figure 6:**
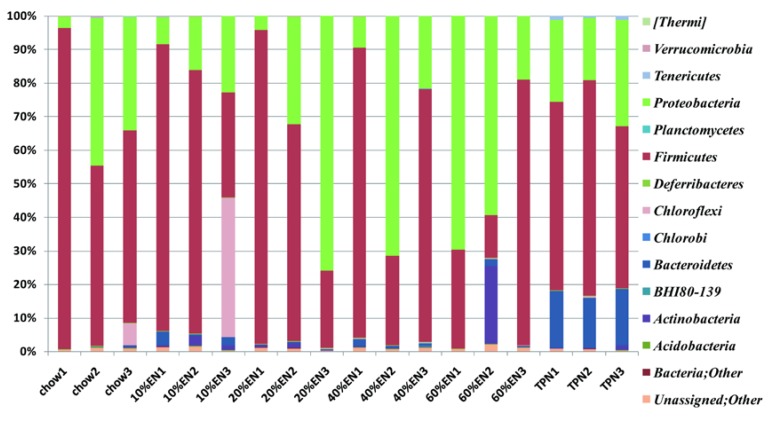
Pyrosequencing analysis of ileal wash samples at the phylum level. EN: enteral nutrition; TPN: total parenteral nutrition.

**Figure 7:**
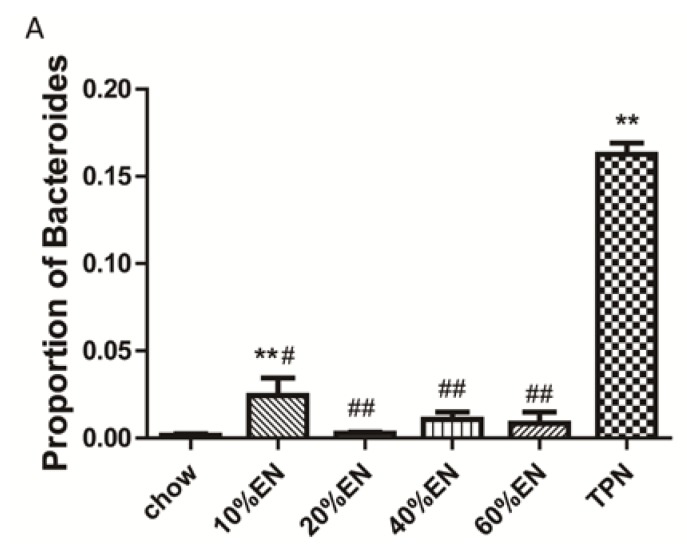

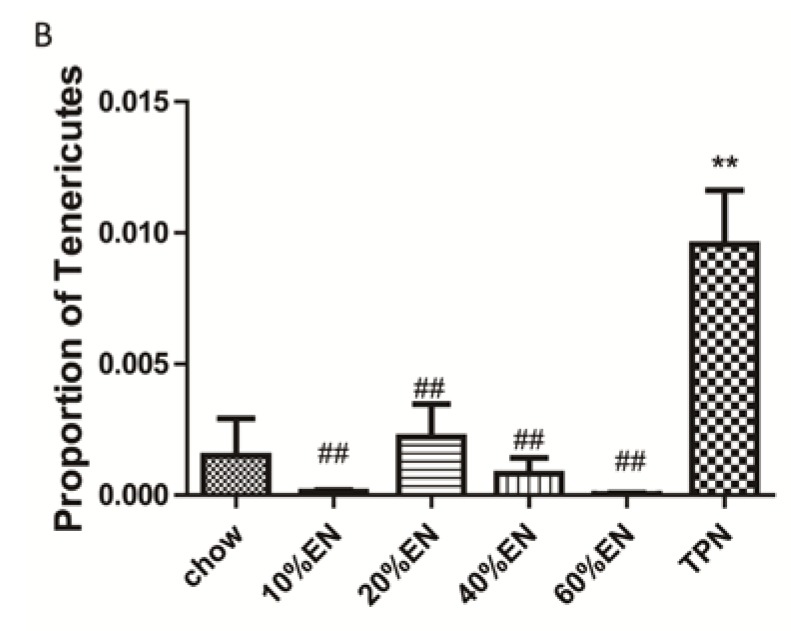
Proportion of Bacteroidetes and Tenericutes in the pyrosequencing analysis of ileal wash samples at the phylum level. (**A**) Bacteroidetes and (**B**) Tenericutes. Data are represented as the mean ± standard error of the mean (SEM). ** *p* < 0.001 *vs.* chow. ^##^ *p* < 0.001 *vs.* TPN. EN: enteral nutrition; TPN: total parenteral nutrition.

16S rRNA pyrosequencing was performed to assess changes in the ileum microbiota in each group. In total, 2,932,800 filtered reads were obtained from the chow (466,254), 10% EN (470,883), 20% EN (325,621), 40% EN (580,420), 60% EN (511,710), and TPN (577,912) groups (Table S4). The microbial composition was analyzed at the phylum level. Rarefaction curves are shown in Figure S1. Although the diversity of each sample varied, the curves were smooth and flattened. The numbers of OTUs were always >1000. The Chao1, Shannon, and Simpson indices (Figure S2, Figure S3, Figure S4) indicated that the sequencing quantity of each sample was capable of sufficiently describing the composition of bacteria. As shown in Figure 6 and Figure 7, the microbiota in the TPN group contained a higher percentage of Bacteroidetes (*p* < 0.001) and Tenericutes (*p* < 0.001) than those in the chow group. The percentage of Bacteroidetes in the 10% EN group was lower than that in the TPN group (*p* < 0.001), but still higher than the chow group (*p* < 0.05). With an EN dose of 20%, the observed percentage of Bacteroidetes became similar to that in the chow group, while it was significantly lower than those in TPN and 10% EN (*p* < 0.05 for each group). The percentage of Tenericutes in microbiota was effectively maintained when partial EN was initiated compared with the chow group, and the amount of EN or chow seldom impacted on the percentage of Tenericutes. However, the percentage of Firmicutes and Proteobacteria did not change significantly when a dose of EN was administered to mice. Detailed results can be found in Table S5. We also analyzed microbial composition at the genus level, but were unable to identify any meaningful results.

### 3.10. Regression Analysis

**Figure 8:**
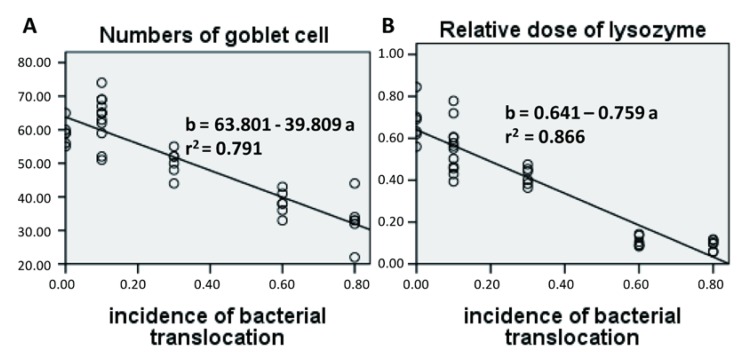

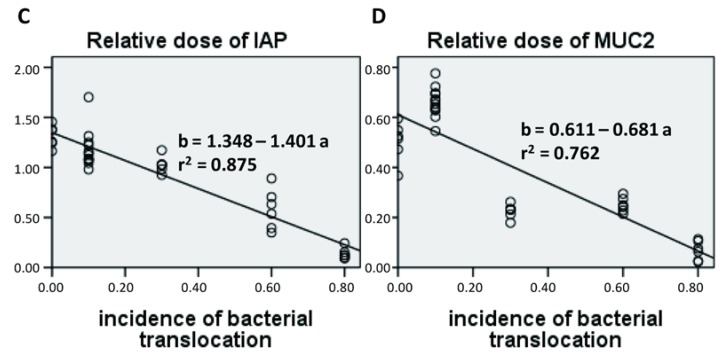
Regression analysis of bacterial translocation and some other defensive components. (**A**) Number of goblet cells; (**B**) Relative dose of lysozyme; (**C**) Relative dose of intestinal alkaline phosphatase (IAP); (**D**) Relative dose of mucin 2 (MUC2). *n* = 36 for each analysis.

The relationships between the bacterial culture and other variables were evaluated using regression analysis, and significant correlations are established in terms of number of goblet cell (*r*^2^ = 0.791, *p* < 0.001), relative lysozyme expression (*r*^2^ = 0.866, *p* < 0.001), relative IAP expression *(r*^2^ = 0.875, *p* < 0.001) and relative MUC2 expression (*r*^2^ = 0.762, *p* < 0.001). All the significant results are shown in Figure 8. However, no linear or quadratic effect was observed in regard to the percentage of intestinal microbiota and other components.

## 4. Discussion

Several clinical studies have established that PN is associated with a high incidence of infection and poor outcomes [1,2,3]. It impairs the function of the intestinal barrier and changes gut flora, which provides a cogent explanation for the observed clinic outcomes [4,5,6,7,12,30,31]. The intestinal barrier comprises a variety of defense mechanisms, including the physical barrier formed by the epithelial cells interconnected with tight junctions, gut immunity comprising acquired immunity and innate immunity, the intestinal biological barrier, and IAP (which interacts with first three barriers). Previous experiments have revealed that these barriers exhibit varying degrees of dysfunction when PN is administered without enteral stimulation. Although the use of EN can reverse this dysfunction, high incidences of vomiting and diarrhea abolish the beneficial effects of high-dose EN therapy in the initial phase [32,33]. Whether a smaller dose of EN has similar benefits has yet to be revealed. Supplementation with a small amount of EN has been shown to improve epithelial cell morphology and acquired immunity during PN in experimental studies [22,23,24]. However, the optimal dose of EN for recovery of gut function remains controversial [19,20,21,22,23,24,34]. In addition, the effects of partial EN on innate immunity, IAP levels, and intestinal microbiota are unknown. To the best of our knowledge, this is the first study to investigate these effects.

Our analysis of the effects of TPN on the intestinal barrier revealed that TPN induced significant impairment of innate immunity, including decreased lysozyme and MUC2 levels. IHC analysis also confirmed that the changes in lysozyme levels were associated with intracellular granules rather than secretory cells. In contrast, changesin MUC2 levels were related to variation in the number of goblet cells. These results are consistent with those of previous reports [5,7]. In addition, PN induced a reduction in IAP levels in the ileum and changed the composition of ileum microbiota, and thereby produced dysfunction of the intestinal barrier [8,9]. We note that administration of TPN increased the percentage of Bacteroidetes and Tenericutes compared with levels observed in chow-fed mice, and this result is in contrast to the previous findings of Heneghan *et al.* [5]. However, in their study, Heneghan *et al.* obtained only 17,830 filtered reads in total. Miyasaka *et al.* [35] reported that Firmicutes made up the vast majority of mucosally associated bacteria in control mice, whereas Proteobacteria and Bacteroidetes were the dominant phyla in the TPN group. However, Miyasaka *et al.* examined the mucosa-associate bacterial population, and our experiment focused on the microbiota in the luminal flushes. Therefore, TPN might exhibit different effects on the microbiota in different locations, and different species of mice can also be another affecting factor. This should be analyzed in detail in future studies.

The present study revealed that various gut barriers were preserved with the administration of EN in a dose-dependent manner, although the observed trends differed somewhat between barriers. Among the parameters examined, only the percentage of Tenericutes decreased in the 10% EN group compared with the TPN group. Supplementation with 20% EN (1) increased the tissue levels of lysozyme and IAP to those observed in the chow group; (2) partially enhanced MUC2 levels and the quantity of goblet cells in the ileum; and (3) decreased the percentage of Bacteroidetes in the intestinal microbiota. Higher doses of EN effectively increased the therapeutic effect in terms of goblet cell numbers (40% EN) and MUC2 levels (60% EN). Conour *et al.* [36] previously demonstrated that administration of PN increased goblet cells in neonatal piglets compared to treatment with EN; however, neonatal animals have considerably different adaptive immune cell compartments compared with adult animals. Our research findings suggest that intestinal barriers are preserved by the administration of EN in a dose-dependent manner, and that all aspects of the intestinal barrier are preserved only when the dose of EN reaches 60%. These conclusions are in agreement with the 2009 European Society of Parenteral and Enteral Nutrition (ESPEN) guidelines, which state that “Combinations of enteral and parenteral nutrition should be considered in patients in whom there is an indication for nutritional support and in whom >60% of energy needs cannot be met via the enteral route”. However, we believe this is the first study to demonstrate the effects of different doses of EN on innate immunity, IAP levels, and gut flora.

Here, we developed a tissue bacterial culture method to investigate incidence of bacterial translocation following PN and EN. We found that, consistent with previous reports, PN significantly increased the incidence of enteroinvasion to the lymph nodes. A 20% dose of EN significantly attenuated bacterial translocation, which suggests that this dose is sufficient to prevent PN associated infections. Therefore, even when patients can tolerate only small amounts of EN, it is likely that treatment will improve gut barrier function to some extent and possibly reduce infection levels. Gut permeability has been used as the standard marker for evaluating intestinal health, and several other investigators have reported that rats fed 15% of their total calorific intake via the enteral route exhibit preserved immunity [20,35]. However, Sax *et al.* [22] demonstrated that 25% EN did not improve gut permeability in a rat feeding model. Therefore, the use of gut permeability as a marker is controversial. In our opinion, incidence of bacterial translocation may be a more reliable marker for estimating the risk of infection.

The intestinal microbiota (which also functions as a biological barrier) plays an important role in maintaining the other intestinal barriers. Lack of an intestinal microbiome is associated with increased risk of infection and susceptibility to enteric pathogens, which suggests that stimulation of intestinal microbiota is an essential component of the host defense mechanism [37]. In this study, we identified the strains present in the bacterial culture in a random manner, and the results revealed that the majority of translocated bacteria were aerobic bacteria rather than an aerobic bacteria (Bacteroidetes). This suggests that most of the cultured bacteria occurred as a result of secondary translocations after intestinal barrier impairment. A recent study reported that Bacteroidetes are resilient to host inflammation and antimicrobial peptides because they encode *LpxF* which is an unusual phosphatidylglycerophosphatase B (PgpB) homolog [38]. Because maintenance of gut barrier function requires stimulation by pathogens and antigens, an increase in the percentage of Bacteroidetes might decrease this stimulation and result in impairment of the gut barrier and secondary infection of intestinal microbiota. Further studies are required to investigate this hypothesis and the general relationship between microbiota and gut barrier function.

We acknowledge that our study has some limitations. Only three mice per group were included in the microbiota identification analysis, and this might explain the negative results obtained for certain microbial phyla, e.g., Firmicutes and Proteobacteria. However, the presence of Firmicutes and Proteobacteria in samples was not linear relating to the dose of EN, which suggests that these phyla were probably not the main bacterial species responding to enteral stimulation. In addition, our statistically significant results indicated that intestinal Bacteroidetes have important effects during PN.

In conclusion, we believe that patients should be administered with a low dose of EN as early as possible in their treatment, as recommended by the nutrition guidelines. Our findings suggest that administration of even 20% EN might provide benefits by enhancing the level of IAP, partially protecting innate immunity, and restoring the composition of intestinal microbiota. Furthermore, the therapeutic effects of EN appear to increase in a dose-dependent manner. Our study provides insights into the mechanisms underlying the protective effects of EN during combined treatment with PN. Partial administration of EN with PN apparently reduces the impact of dysfunctional gut barriers and improves clinical outcomes in critical illnesses. 

## 5. Conclusions

Supplementation of PN with 20% EN preserves gut barrier function, by way of maintaining innate immunity, IAP and intestinal microbiota.

## Supplementary Files

Supplementary File 1
