# Supplementary material for: Partial Enteral Nutrition Preserves Elements of Gut Barrier Function, Including Innate Immunity, Intestinal Alkaline Phosphatase (IAP) Level, and Intestinal Microbiota in Mice
Source: Nutrients. 2015 Aug 3;7(8):6294–312. doi: 10.3390/nu7085288 (PMC4555127; doi:10.3390/nu7085288)
Supplement: Supplementary File 1 [file nutrients-07-05288-s001.docx]

**Supplementary Information**

**Table S1.** The data of goblet cell numbers.

|  | **Chow** | **10% EN** | **20% EN** | **40% EN** | **60% EN** | **TPN** |
| --- | --- | --- | --- | --- | --- | --- |
| Goblet cell numbers | 61.6 ± 6.47 | 38.2 ± 3.96 | 50.2 ± 4.27 | 59.0 ± 3.94 | 65.2 ± 8.61 | 33.0 ± 7.81 |
| Compared with chow |  | *p* < 0.001 | *p* *=* 0.007 | *p* *=* 0.510 | *p =* 0.363 | *p* < 0.001 |
| Compared with TPN | *p* < 0.001 | *p* *=* 0.193 | *p* < 0.001 | *p* < 0.001 | *p* < 0.001 |  |

EN: enteral nutrition; TPN: total parenteral nutrition.

**Table S2.** All the results of protein levels.

|  | **Chow** | **10% EN** | **20% EN** | **40% EN** | **60% EN** | **TPN** |
| --- | --- | --- | --- | --- | --- | --- |
| Lysozyme | 0.4786 ± 0.0636 | 0.1085 ± 0.0261 | 0.4199 ± 0.0486 | 0.6748 ± 0.1502 | 0.6291 ± 0.1395 | 0.0909 ± 0.0289 |
| Compared with chow |  | *p* < 0.001 | *p =* 0.446 | *p =* 0.022 | *p* *=* 0.066 | *p* < 0.001 |
| Compared with TPN | *p* < 0.001 | *p =* 0.818 | *p =* 0.001 | *p* < 0.001 | *p* < 0.001 |  |
| MUC2 | 0.6729 ± 0.1171 | 0.2506 ± 0.0230 | 0.2254 ± 0.0114 | 0.5039 ± 0.1209 | 0.6494 ± 0.0194 | 0.0679 ± 0.0437 |
| Compared with chow |  | *p* < 0.001 | *p* < 0.001 | *p* *=* 0.014 | *p =* 0.698 | *p* < 0.001 |
| Compared with TPN | *p* < 0.001 | *p =* 0.009 | *p =* 0.020 | *p* < 0.001 | *p* < 0.001 |  |
| IAP | 1.0812 ± 0.0732 | 0.5848 ± 0.2575 | 1.0268 ± 0.1053 | 1.3131 ± 0.1286 | 1.2822 ± 0.2897 | 0.1390 ± 0.0705 |
| Compared with chow |  | *p =* 0.001 | *p =* 0.669 | *p =* 0.081 | *p =* 0.126 | *p* < 0.001 |
| Compared with TPN | *p* < 0.001 | *p =* 0.002 | *p* < 0.001 | *p* < 0.001 | *p* < 0.001 |  |

EN: enteral nutrition; TPN: total parenteral nutrition; IAP: intestinal alkaline phosphatase; MUC2: mucin2.

**Table S3.** All the results of mRNA expressions.

|  | **Chow** | **10% EN** | **20% EN** | **40% EN** | **60% EN** | **TPN** |
| --- | --- | --- | --- | --- | --- | --- |
| Lysozyme | 0.6407 ± 0.2827 | 0.2122 ± 0.2208 | 0.5588 ± 0.8461 | 0.7192 ± 0.5369 | 0.6110 ± 0.3534 | 0.2074 ± 0.2214 |
| Compared with chow |  | *p =* 0.014 | *p =* 0.0632 | *p =* 0.646 | *p =* 0.862 | *p =* 0.013 |
| Compared with TPN | *p =* 0.013 | *p* = 0.977 | *p* = 0.042 | *p =* 0.004 | *p* = 0.020 |  |
| MUC2 | 0.9356 ± 0.1350 | 0.3253 ± 0.0535 | 0.3817 ± 0.0852 | 0.6758 ± 0.1050 | 0.9997 ± 0.2293 | 0.1723 ± 0.0635 |
| Compared with chow |  | *p* <0.001 | *p* <0.001 | *p* < 0.001 | *p* = 0.349 | *p* < 0.001 |

**Table S3.** *Cont.*

|  | **Chow** | **10% EN** | **20% EN** | **40% EN** | **60% EN** | **TPN** |
| --- | --- | --- | --- | --- | --- | --- |
| Compared with TPN | *p* < 0.001 | *p =* 0.03 | *p =* 0.004 | *p* < 0.001 | *p* < 0.001 |  |
| IAP | 0.6588 ± 0.5940 | 0.4255 ± 0.1285 | 0.6975 ± 0.1005 | 0.8476 ± 0.2397 | 0.8443 ± 0.2089 | 0.2368 ± 0.1212 |
| Compared with chow |  | *p =* 0.031 | *p* = 0.975 | *p =* 0.489 | *p* = 0.377 | *p* < 0.001 |
| Compared with TPN | *p* <0.001 | *p =* 0.167 | *p* < 0.001 | *p =* 0.003 | *p =* 0.001 |  |

EN: enteral nutrition; TPN: total parenteral nutrition; IAP: intestinal alkaline phosphatase; MUC2: mucin2.

**Table S4.** Statistics of Raw Data in the pyrosequencing analysis of ileal wash samples.

| **Sample** | **Raw Data** | | **High-Quality Reads & Removed Low Density OTUs** | |
| --- | --- | --- | --- | --- |
|  | **Number of Reads** | **Average Length** | **Number of Reads** | **Average Length** |
| chow1 | 153,439 | 300.11 | 90,519 | 285.15 |
| chow2 | 85,925 | 283.18 | 53,965 | 227.49 |
| chow3 | 489,095 | 292.77 | 321,770 | 258.6 |
| 10% EN1 | 243,944 | 277.66 | 133,229 | 218.28 |
| 10% EN2 | 143,784 | 273.30 | 70,270 | 239.31 |
| 10% EN3 | 373,149 | 313.73 | 267,384 | 283.22 |
| 20% EN1 | 288,658 | 271.72 | 151,576 | 215.88 |
| 20% EN2 | 131,707 | 263.95 | 73,583 | 235.01 |
| 20% EN3 | 165,051 | 302.49 | 100,462 | 284.93 |
| 40% EN1 | 285,149 | 283.38 | 135,544 | 240.3 |
| 40% EN2 | 150,161 | 290.78 | 90,752 | 272.48 |
| 40% EN3 | 517,881 | 300.39 | 354,124 | 264.08 |
| 60% EN1 | 164,325 | 272.35 | 80,085 | 284.36 |
| 60% EN2 | 164,527 | 264.63 | 89,862 | 228.7 |
| 60% EN3 | 496,416 | 301.42 | 341,763 | 271.98 |
| TPN1 | 289,581 | 260.25 | 132,631 | 241.28 |
| TPN2 | 251,614 | 260.28 | 111,611 | 239.1 |
| TPN3 | 497,174 | 291.79 | 333,670 | 263.71 |

EN: enteral nutrition; TPN: total parenteral nutrition; OTUs: operational taxonomic units.

**Table S5.** All the results of 16s RNA Pyrosequencing.

|  | **Chow** | **10% EN** | **20% EN** | **40% EN** | **60% EN** | **TPN** |
| --- | --- | --- | --- | --- | --- | --- |
| Acidobacteria | 0.0039 ± 0.0038 | 0.0342 ± 0.0442 | 0 | 0.0154 ± 0.0191 | 0.0024 ± 0.0018 | 0.0009 ± 0.0009 |
| Compared with chow |  | *p =* 0.084 | *p* = 0.813 | *p =* 0.490 | *p* =0.927 | *p* = 0.853 |
| Compared with TPN | *p =* 0.853 | *p* = 0.061 | *p* = 0.959 | *p* = 0.386 | *p* = 0.926 |  |
| Actinobacteria | 0.1623 ± 0.1714 | 1.5959±1.2232 | 0.7740 ± 0.7063 | 0.3171 ± 0.1448 | 7.8526 ± 13.4070 | 0.5104 ± 0.7825 |
| Compared with chow |  | *p* = 0.756 | *p* = 0.894 | *p* = 0.973 | *p* = 0.113 | *p* *=* 0.940 |
| Compared with TPN | *p* = 0.940 | *p* = 0.814 | *p* = 0.954 | *p* = 0.966 | *p* *=* 0.129 |  |
| BHI80-139 | 0 | 0 | 0 | 0.0089 ± 0.0155 | 0 | 0 |
| Compared with chow |  | *p =* 1 | *p =* 1 | *p =* 0.109 | *p* *=* 1 | *p* *=* 1 |
| Compared with TPN | *p =* 1 | *p =* 1 | *p =* 1 | *p =* 0.109 | *p =* 1 |  |
| Bacteroidetes | 0.1590 ± 0.1370 | 2.4452 ± 1.7128 | 0.2766 ± 0.1147 | 1.0696 ± 0.7297 | 0.8390 ± 1.1406 | 16.2557 ± 1.1444 |
| Compared with chow |  | *p =*  0.017 | *p =* 0.889 | *p =* 0.291 | *p =* 0.425 | *p* < 0.001 |
| Compared with TPN | *p* < 0.001 | *p* < 0.001 | *p* < 0.001 | *p* < 0.001 | *p* <0.001 |  |
| Chlorobi | 0 | 0 | 0 | 0.0176 ± 0.0305 | 0 | 0 |
| Compared with chow |  | *p =* 1 | *p* *=* 1 | *p =* 0.109 | *p =* 1 | *p =* 1 |
| Compared with TPN | *p =* 1 | *p =* 1 | *p* *=* 1 | *p =* 0.109 | *p =* 1 |  |
| Chloroflexi | 2.2805 ± 3.8672 | 13.8538 ± 23.9089 | 0.1290 ± 0.0993 | 0.3626 ± 0.1294 | 0.0191 ± 0.0204 | 0.3111 ± 0.1590 |
| Compared with chow |  | *p =*  0.177 | *p* *=* 0.794 | *p =* 0.816 | *p* *=* 0.784 | *p* *=* 0.811 |
| Compared with TPN | *p =* 0.811 | *p =*  0.119 | *p =* 0.982 | *p =* 0.995 | *p* *=* 0.972 |  |
| Deferribacteres | 0.0094 ± 0.0118 | 0.0424 ± 0.0504 | 0.1073 ± 0.1219 | 0.0112 ± 0.0128 | 0.0802 ± 0.0971 | 0.0751 ± 0.1109 |
| Compared with chow |  | *p* *=* 0.627 | *p* *=* 0.165 | *p =* 0.978 | *p =* 0.306 | *p =* 0.340 |
| Compared with TPN | *p =* 0.340 | *p =* 0.630 | *p =* 0.636 | *p =* 0.353 | *p =* 0.941 |  |
| Firmicutes | 69.4956 ± 22.7673 | 65.1794 ± 29.4378 | 60.3082 ± 35.4670 | 62.8387 ± 41.8780 | 40.4803 ± 34.6113 | 56.1604 ± 7.9276 |
| Compared with chow |  | *p =* 0.857 | *p =* 0.701 | *p =* 0.781 | *p =* 0.238 | *p =* 0.579 |
| Compared with TPN | *p =* 0.579 | *p =* 0.706 | *p =* 0.862 | *p* *=* 0.780 | *p* *=* 0.515 |  |
| Planctomycetes | 0 | 0 | 0 | 0.0366 ± 0.0634 | 0 | 0 |
| Compared with chow |  | *p* *=* 1 | *p* *=* 1 | *p* *=* 0.109 | *p* *=* 1 | *p* *=* 1 |
| Compared with TPN | *p* *=* 1 | *p* *=* 1 | *p* *=* 1 | *p* *=* 0.109 | *p* *=* 1 |  |
| Proteobacteria | 27.6388 ± 21.6954 | 15.5876 ± 7.3495 | 37.4787 ± 36.1928 | 34.1617 ± 32.9655 | 49.3319 ± 26.8041 | 24.9677 ± 6.4932 |
| Compared with chow |  | *p* *=* 0.562 | *p* *=* 0.635 | *p* *=* 0.753 | *p* *=* 0.305 | *p* *=* 0.897 |
| Compared with TPN | *p* *=* 0.897 | *p* *=* 0.651 | *p* *=* 0.548 | *p* *=* 0.658 | *p* *=* 0.252 |  |
| Tenericutes | 0.1509 ± 0.2450 | 0.0212 ± 0.0218 | 0.0770 ± 0.1333 | 0.0818 ± 0.1035 | 0.0044 ± 0.0057 | 0.9554 ± 0.3572 |
| Compared with chow |  | *p* *=* 0.420 | *p* *=* 0.643 | *p =* 0.664 | *p* *=* 0.364 | *p* < 0.001 |
| Compared with TPN | *p* < 0.001 | *p* < 0.001 | *p* < 0.001 | *p* < 0.001 | *p* < 0.001 |  |
| Verrucomicrobia | 0.1443 ± 0.2471 | 0.1369 ± 0.1311 | 0.0005 ± 0.0008 | 0.0010 ± 0.0017 | 0 | 0 |
| Compared with chow |  | *p =* 0.938 | *p =* 0.149 | *p =* 0.150 | *p =* 0.148 | *p =* 0.148 |
| Compared with TPN | *p =* 0.148 | *p =* 0.168 | *p =* 0.996 | *p =* 0.992 | *p =* 1 |  |

EN: enteral nutrition; TPN: total parenteral nutrition.


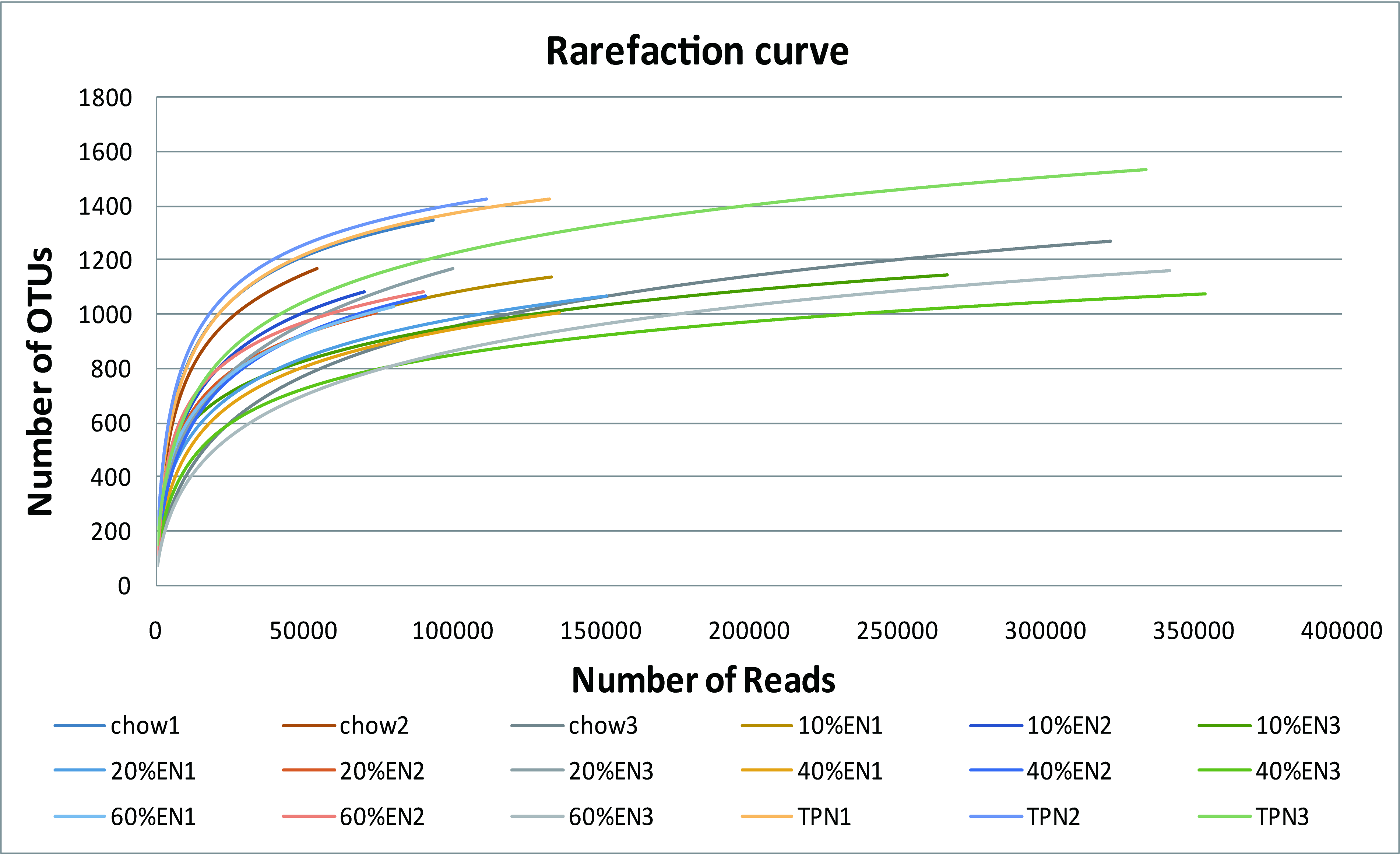


**Figure S1.** Rarefaction curve. EN: enteral nutrition; TPN: total parenteral nutrition; OUTs: operational taxonomic units.


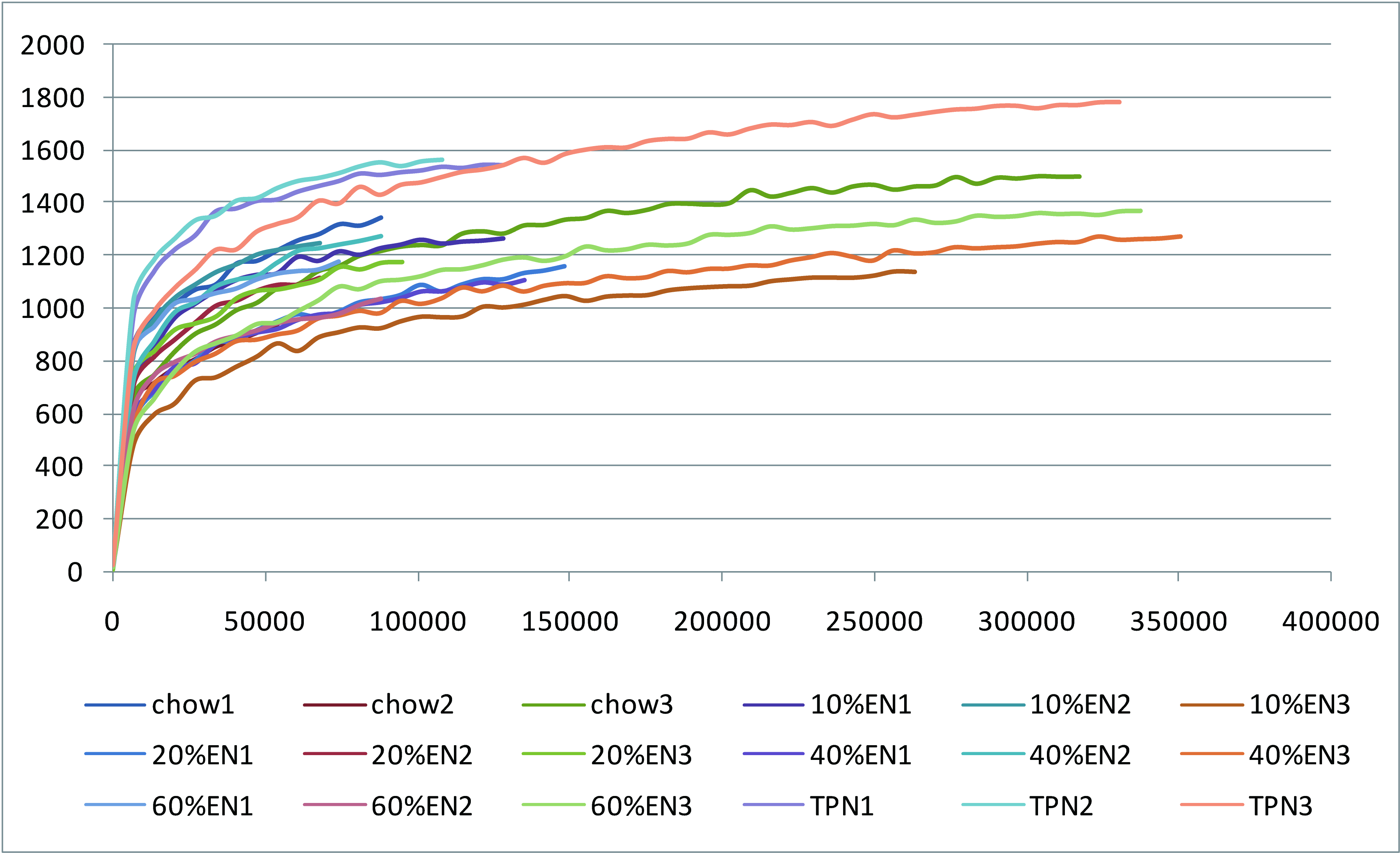


**Figure S2.** Chao1 indices. EN: enteral nutrition; TPN: total parenteral nutrition.


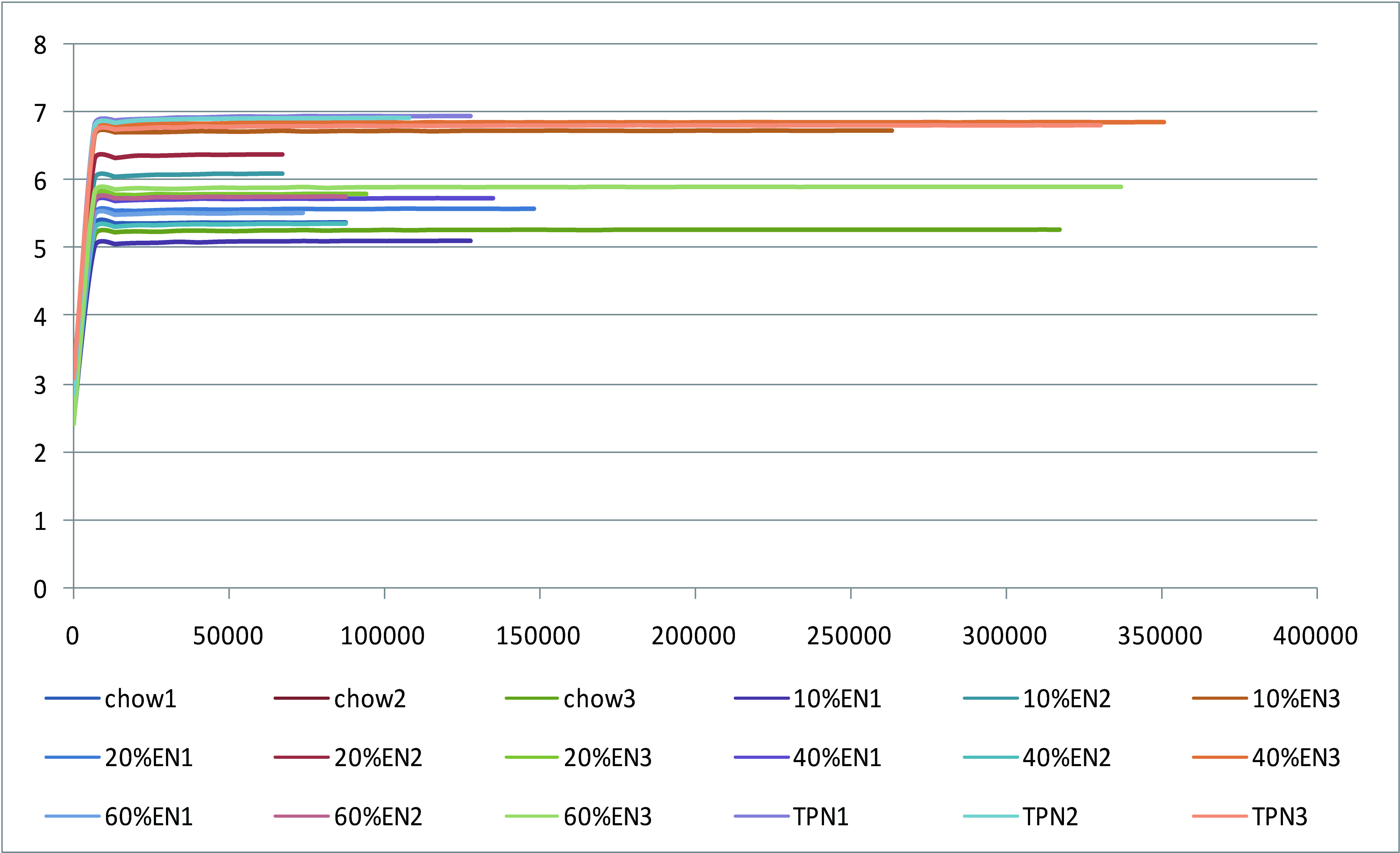


**Figure S3.** Shannon indices. EN: enteral nutrition; TPN: total parenteral nutrition.


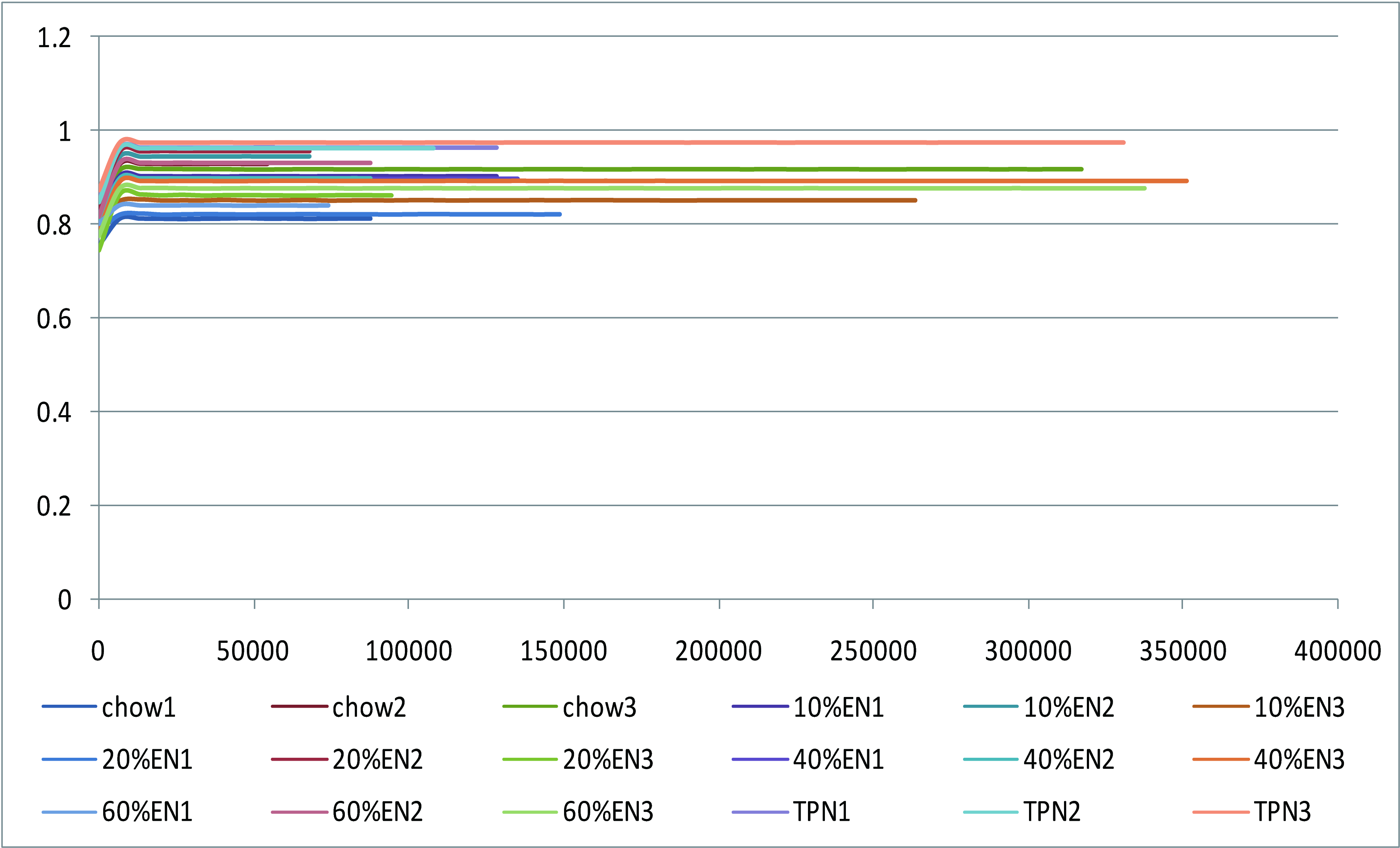


**Figure S4.** Simpson indices. EN: enteral nutrition; TPN: total parenteral nutrition.
